# miRNA-mRNA analysis of sheep adrenal glands reveals the network regulating reproduction

**DOI:** 10.1186/s12863-022-01060-y

**Published:** 2022-06-17

**Authors:** Yulin Chen, Yufang Liu, Mingxing Chu

**Affiliations:** 1 Key Laboratory of Animal Genetics, Breeding and Reproduction of Ministry of Agriculture and Rural Affairs, Institute of Animal Science, Chinese Academy of Agricultural Sciences, Beijing, 100193 China; 2 College of Life Sciences and Food Engineering, Hebei University of Engineering, Handan, 056021 China

**Keywords:** DEMs, Follicular phase, Luteal phase, Adrenal glands, Sheep

## Abstract

**Background:**

The adrenal gland participates in the process of sheep reproduction. MicroRNAs (miRNAs), endogenous small noncoding RNAs, regulate gene expression at the posttranscriptional level. However, the miRNA-mRNA network profile of adrenal glands relating to reproduction in sheep is still not well-studied. As sheep with FecB^BB^ genotype show higher lambing number compare with the sheep with FecB^++^ genotype. This research aims to compare gene expression by small RNA-seq in adrenal tissues at follicular (F) and luteal (L) phases in *FecB*^BB^ (MM) and *FecB*^++^ (ww) sheep. After analysis of gene expression, significant differentially expressed microRNAs (DEMs) and corresponding target genes were identified.

**Results:**

A total of 180 miRNAs were found in this study, of which 19 DEMs were expressed in the four comparison groups (MM_F_A vs. MM_L_A, MM_F_A vs. ww_F_A, MM_L_A vs. ww_L_A, ww_F_A vs. ww_L_A). Subsequently, 354 target genes of 19 DEMs were predicted by integrated analysis. Cluster analysis was performed by K_means_cluster, and the expression patterns of these DEMs were separated into four subclusters. Functional analysis of target genes was performed by Gene Ontology (GO) and Kyoto Encyclopedia of Genes and Genomes (KEGG). The results indicated that the target genes were involved mainly in the Notch signaling pathway, signal transduction, cell communication, innate immune response and amino acid metabolism. Specifically, the Notch signaling pathway, biosynthetic process and metabolic process of pyrimidine nucleotide and amino acid metabolism appear to play key regulatory roles in the sheep fertility trait. Furthermore, miRNA-mRNA interaction networks were constructed by differentially expressed genes combined with our previous study of transcriptome data. The results showed that several key genes, including *TDRD3*, *ANAPC7*, *CCNL2*, *BRD2* and *MUT,* were related to the transformation from the follicular phase to the luteal phase. *PLAC8L1*, *NFAT5*, *DDX24* and *MBD1* were related to the high fecundity of small tail Han sheep.

**Conclusions:**

In this study, the miRNA transcriptome profile was identified, and miRNA-mRNA interaction networks were constructed in adrenal gland tissue of small tail Han sheep, the interaction between miR-370-3p and its targets were considered to play a major role in the reproduction regulation process. The results enriched the number of known miRNAs in adrenal glands and provided novel ideas and further information to demonstrate posttranscriptional regulation mechanisms at follicular and luteal phases in different genotypes of small tail Han sheep.

**Supplementary Information:**

The online version contains supplementary material available at 10.1186/s12863-022-01060-y.

## Background

Small ruminants, particularly native breeds, play a significant role in the livelihoods of a considerable part of the human population from socioeconomic aspects [1]. Thus, combined trials with emphasis on administration and genetic progress to improve animal outputs are of decisive significance [2]. The economic and biological efficiency of sheep production enterprises generally improves by increasing the productivity and reproductive performance of ewes [3, 4]. Sheep is one of the most important agricultural animals [5]. Small tail Han sheep are famous in China for their reproductive traits, such as high fertility, growth speed, high-quality coat and body shape [6]. The behavior of estrus and mating in small tail Han sheep occurs year-round. The lambing rate of primiparous ewes is approximately 200%, and in produced ewes, it is higher than 250% [7]. The adrenal gland is one of the tissues participating in the activity controlling the estrus phase, and study has confirmed the relationship between estrus suppression and adrenal function [8]. Besides, considerable evidence indicates that adrenal hormones may affect gonadal function. Study has shown that adrenal gland and adrenal-mediated urinary metabolites play an important role in the estrus suppression [9]. Locally increased cortisol level may serve to minimise inflammatory tissue damage and accelerat ovulatory cycle [10]. And glucocorticoids which are broadly assumed to have a negative impact on reproductive function now were found also play active role [11]. Sheep with FecB mutations have more ovulation and lamb numbers, which produce huge economic benefits in production [12]. According to a previous study, all three genotypes of FecB (FecB BB, FecBB+ and FecB++) are distributed in small tail Han sheep, and the three different genotypes of FecB have a significant correlation with the litter size of ewes [13]. However, the variation in the transcriptome in adrenal glands at different genotypes of FecB is still unclear.

Moreover, the epigenome comprising different mechanisms, e.g., DNA methylation, remodeling, histone tail modifications, chromatin microRNAs and long noncoding RNAs, interacts with environmental factors such as nutrition, pathogens, and climate to influence the expression profile of genes and the emergence of specific phenotypes [14]. Multi-level interactions between the genome, epigenome and environmental factors might occur. Furthermore, numerous lines of evidence suggest the influence of epigenome variation on health and production [15]. As a type of 22 nucleotide (nt)-long endogenous small noncoding RNA, microRNAs (miRNAs) can regulate gene expression at the posttranscriptional level in organisms [16]. miRNAs play a pivotal role in many biological processes (development, metabolism, differentiation, etc.) and have become a hot research topic in genetics. A previous study of sheep ovaries showed that the DEMs and their target mRNAs would help to explore the molecular regulatory mechanisms and screen biomarkers that affect ovarian development [17]. Bai et al. identified 153 known sheep miRNAs and 2712 novel miRNAs from testes and screened out the key miRNA targets involved in testis development and spermatogenesis [18]. As these studies showed, miRNAs participate in regulating reproductive processes in the testes and ovaries of sheep. Studies have shown that the adrenal glands also have an impact on reproduction, and previous studies have shown that glucocorticoids secreted by adrenal glands can impair oocyte developmental potential. In follicular fluid, the levels of cortisol could be used as an index of fertilization. Research in sheep has demonstrated that glucocorticoids acting on the hypothalamus-pituitary–gonadal (HPG) axis could decrease gonadotrophin secretion and correspondingly reduce the responsiveness and sensitivity of gonadotrophic cells and their receptors to GnRH [19–23]. However, studies on the functions of miRNAs in this organ are limited. Therefore, to further investigate the regulatory mechanism of miRNAs in adrenal glands, we identified the miRNAs and constructed miRNA-mRNA interaction networks during the follicular phase and luteal phase of sheep of different genotypes.

In this study, small RNA-seq was used to detect the significantly differentially expressed miRNAs in the four groups. Target genes of miRNAs were predicted by three software programs, and Gene Ontology (GO) and the Kyoto Encyclopedia of Genes and Genomes (KEGG) analyses of these target genes were conducted. Several genes responsible for reproduction were screened after analysis, which may provide a further understanding of the interaction between the HPG and hypothalamus–pituitary–adrenal (HPA) axes. In addition, the lncRNA and circRNA data acquired from small RNA-seq were also processed and analyzed. As the different mechanism and influence among miRNA, lncRNA and circRNA, the methods and analysis were different.. Therefore, the corresponding study of different RNAs was discussed and reported independently [24].

## Results

### Small-RNA library sequencing and quality control

The small RNA-seq data of 12 samples (according to different FecB (BB and + +) genotypes and different stages at the estrus cycle divided into 4 groups and 3 individuals in each group, named MM_F_A (FecBBB at follicular phase), MM_L_A (FecBBB at luteal phase), ww_F_A (FecB++ at follicular phase) and ww_L_A (FecB++ at luteal phase), respectively) were subjected to quality control, and the Q30 values ranged from 93.14% to 96.48%. The results are shown in Table 1. After adapter trimming and quality checks, we obtained 10 to 14 million clean reads from these samples. A length filter was used to obtain the type and number of sRNAs for the next analysis. A range of 85.60% to 90.87% of the total sRNA was mapped to the sheep reference genome. miRNA identification of these mapped sequences was subsequently performed.

**Table 1:** Summary of quality data after quality control

Sample	Raw reads	Q30(%)	Clean reads Clean reads rate (%)	Total sRNA	Total mapped reads
MM_F_A_1	12,889,364	96.43%	12,521,753 (97.15%)	11,309,966	10,277,206(90.87%)
MM_F_A_2	12,849,723	96.48%	12,647,334 (98.42%)	11,516,296	10,400,403(90.31%)
MM_F_A_3	12,081,611	94.62%	11,818,112 (97.82%)	10,404,956	9,105,628 (87.51%)
MM_L_A_1	12,206,638	93.70%	11,994,124 (98.26%)	10,906,828	9,722,144 (89.14%)
MM_L_A_2	13,159,620	93.48%	12,918,303 (98.17%)	11,018,323	9,857,239 (89.46%)
MM_L_A_3	11,494,399	95.37%	11,321,359 (98.49%)	10,395,332	9,016,066 (86.73%)
ww_F_A_1	13,300,254	94.30%	13,033,005 (97.99%)	11,259,335	9,755,237 (86.64%)
ww_F_A_2	12,758,993	94.43%	12,545,850 (98.33%)	11,497,406	10,090,539(87.76%)
ww_F_A_3	14,796,591	93.14%	14,465,181 (97.76%)	13,126,840	11,522,684(87.78%)
ww_L_A_1	10,877,209	95.03%	10,499,033 (96.52%)	9,553,427	8,232,140 (86.17%)
ww_L_A_2	12,403,470	95.33%	12,180,506 (98.20%)	11,422,289	10,046,316(87.95%)
ww_L_A_3	12,068,398	95.21%	11,771,700 (97.54%)	10,158,259	8,695,733 (85.60%)

*Note*: MM_F_A and MM_L_A represent follicular phase and luteal phase in MM sheep, respectively; ww_F_A and ww_L_A represent follicular phase and luteal phase in ww sheep, respectively. The same as below

### miRNAs expressed in adrenal gland tissues

After length filtering, known miRNA analysis was performed by comparison with the specified range sequence in miRbase. The range from 3 076 452 to 5 122 424 unique sequences (48 712 659 total reads) was completely matched to the miRNAs of the sheep. Structural predictions of precursor sequences were conducted, and 149 known miRNAs and 106 hairpin miRNAs were identified in adrenal gland tissue. After elimination of the tRNA, rRNA, snoRNA, and other snRNA sequences, the comparison between the remaining sequences and the mature sequence of miRNAs from sheep in miRbase was then performed. As a result, 31 mature novel miRNAs and 32 novel miRNAs were predicted by the iconic hairpin structure of the precursor of miRNA (shown in Table 2). Additionally, family analysis of the identified miRNAs showed that 111 miRNA precursors belonged to 57 miRNA gene families. During miRNA families, up to 10 families contained more than two precursors, and most members of these families had higher abundance in sheep adrenal gland tissue. As the most abundant miRNA family, 19 members of the miR-154 family were detected in sheep adrenal gland tissue. The miRNA families of miR-30, let-7, miR-368, miR-10, miR-379 and miR-329 had 4, 7, 5, 4, 7 and 4 miRNA precursors, respectively.

**Table 2:** Statistics for the miRNAs identified in each library

Types	total	MM_F_A_1	MM_F_A_2	MM_F_A_3	MM_L_A_1	MM_L_A_2	MM_L_A_3
Known miRNA							
Mapped mature	149	123	143	135	139	140	144
Mapped hairpin	106	102	106	104	104	103	106
Mapped uniq sRNA	21,719	1538	1827	1605	1799	1845	2012
Mapped total sRNA	48,712,659	4,101,801	5,122,424	4,246,665	4,190,025	4,165,253	3,703,148
Novel miRNA							
Mapped mature	31	16	13	19	17	10	13
Mapped hairpin	32	17	14	19	18	12	14
Mapped uniq sRNA	542	63	42	44	41	39	45
Mapped total sRNA	11,805	672	961	872	1359	1286	885
Known miRNA							
Mapped mature	149	138	139	146	140	141	142
Mapped hairpin	106	103	104	106	104	104	106
Mapped uniq sRNA	21,719	1704	1650	2152	1777	1814	1996
Mapped total sRNA	48,712,659	3,912,104	3,941,907	4,492,611	3,230,607	4,529,662	3,076,452
Novel miRNA							
Mapped mature	31	17	14	14	15	16	15
Mapped hairpin	32	17	15	15	16	17	19
Mapped uniq sRNA	542	46	37	45	44	49	47
Mapped total sRNA	11,805	947	692	1315	1218	936	662

### DE miRNAs in adrenal gland tissue

**Fig. 1:**
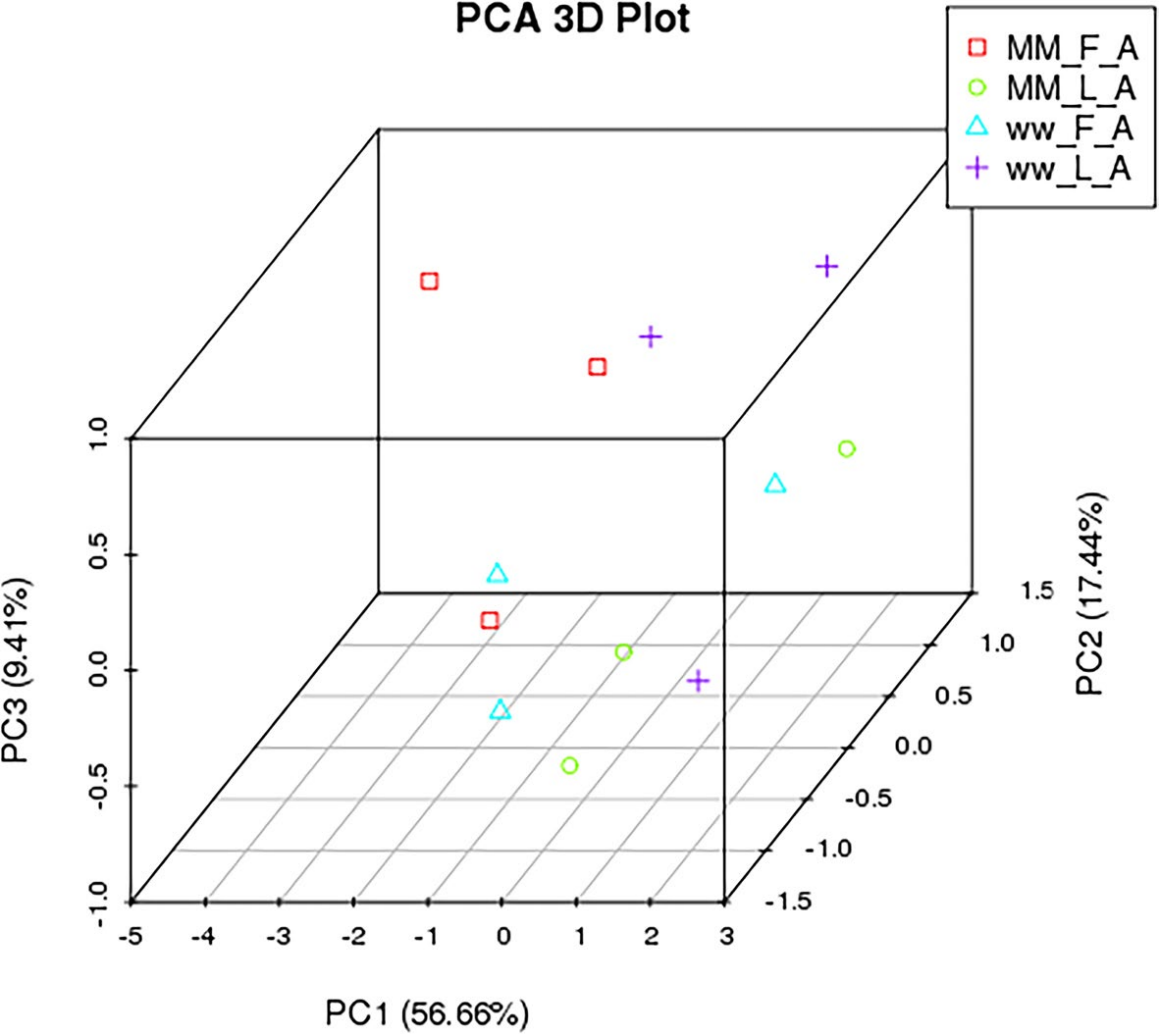
principal component analysis of the differentially expressed miRNAs

**Fig. 2:**
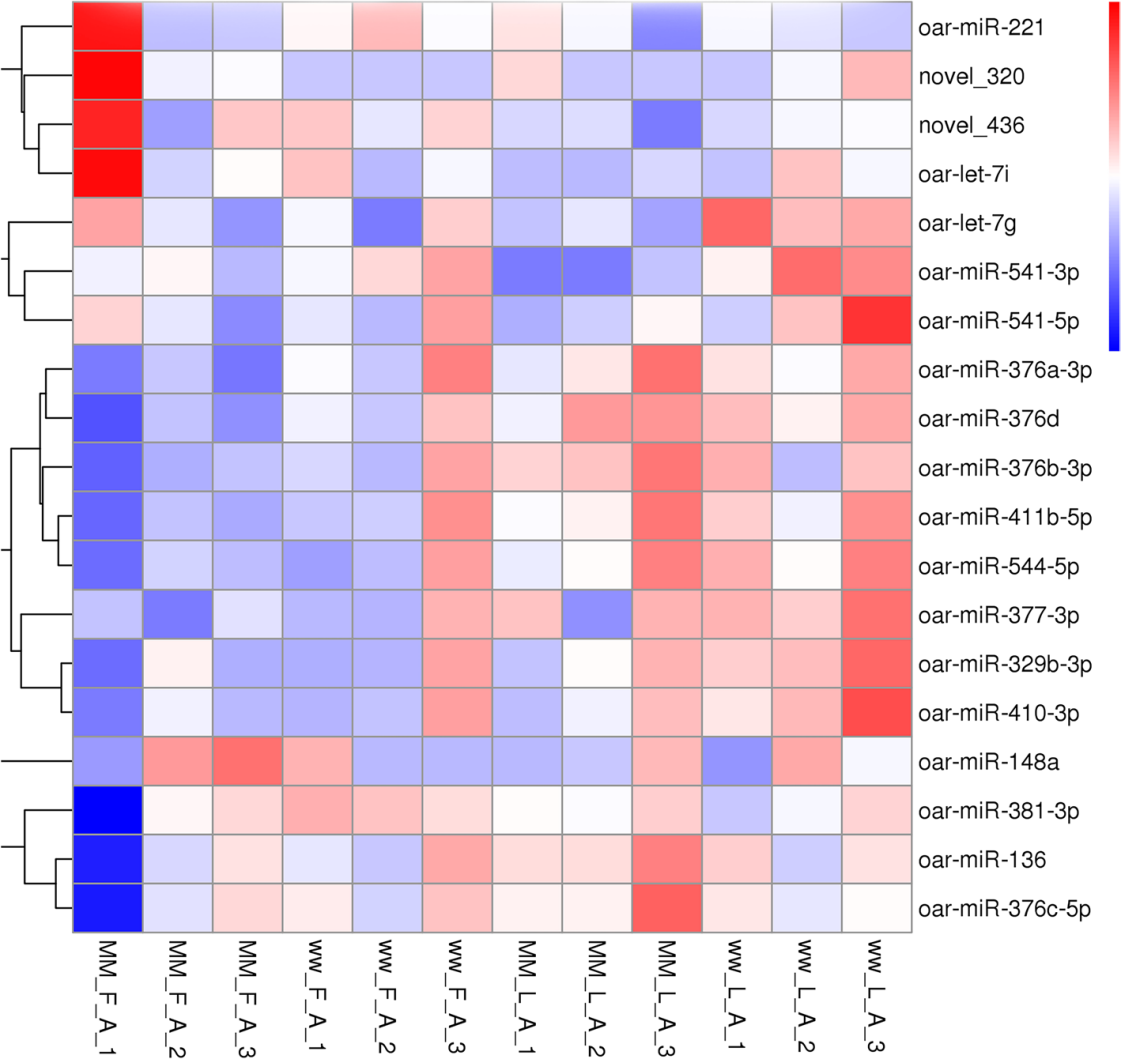
Cluster analysis of differentially expressed sRNA. Red indicates high expression and blue indicates low expression. The color is from red to blue, indicating log10 (TPM + 1) from large to small

To determine the similarities of samples, unsupervised hierarchical clustering and principal component analysis were performed for all the differentially expressed miRNAs (|FC|> 1, without considering the FDR and mean read count)(Fig. 1, Fig. 2). The average percentages of miRNAs with TPM > 60 in MM_F_A, MM_L_A, ww_F_A and ww_L_A were 35.56%, 44.63%, 41.29% and 46.11%, respectively, which suggests the higher expression abundance of miRNAs in the luteal phase than in the follicular phase. As the heatmap and clustering tree shows, the miRNA expression pattern were consistent within group, and the miRNA expression pattern of different comparison groups were distinct different. The miRNA expression pattern found by heatmap and clustering tree showed a distinct difference during the comparison groups, within each group, the expression patterns of miRNAs were consistent enough, which could distinguish the different comparison groups easily. The data indicated that there were significant changes from the follicular phase to the luteal phase in the miRNA profile in adrenal glands.

**Fig. 3:**
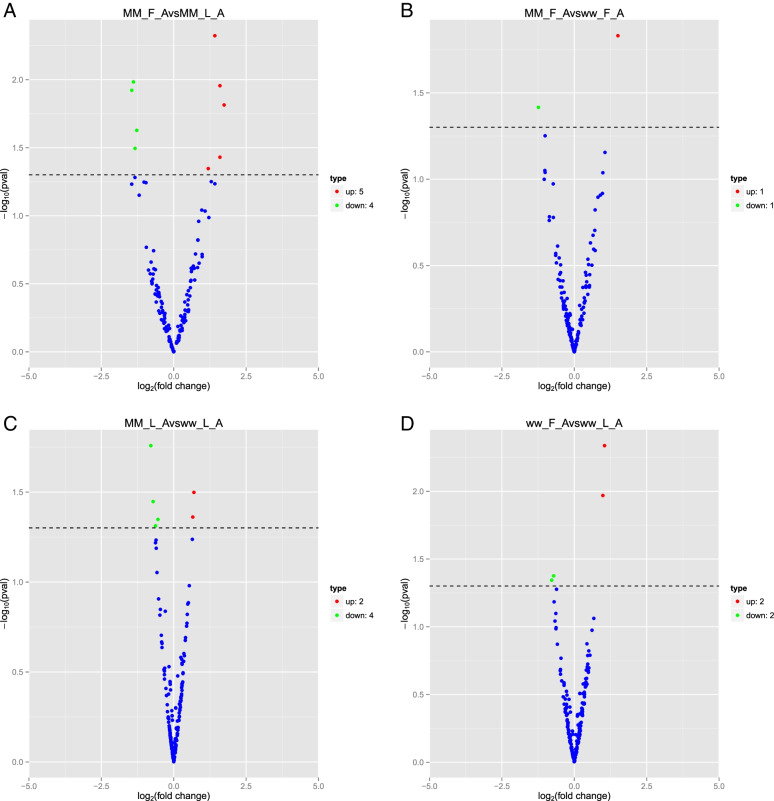
Volcano plot of DE miRNAs. Horizontal coordinates represent miRNA expression fold changes in different experimental groups/different samples, vertical coordinates represent the statistical significance of miRNA expression changes, scattered dots in the graph represent individual miRNAs, blue dots indicate miRNAs with no significant differences, red dots indicate significantly up-regulated differential miRNAs, green dots indicate significantly down-regulated differential miRNAs

The expression levels of the identified miRNAs in the four comparison groups were compared. Under the criteria of a Q-value < 0.01 and | log2(fold change) |> 1, a total of 19 DEMs (9 up- and 10 downregulated) were obtained from the MM_L_A vs. ww_L_A, MM_F_A vs. ww_F_A, MM_F_A vs. MM_L_A and ww_F_A vs. ww_L_A comparison groups. In particular, there were 9 DEMs (4 down- and 5 upregulated) in the MM_F_A vs. MM_L_A group and 6 DEMs (4 down- and 2 upregulated) in the MM_L_A vs. ww_L_A group(Fig. 3). These DEMs were speculated to have a certain effect in adrenal glands and influenced the fertility trait of small-tailed Han sheep.

### Target gene predictions and functional analysis

**Fig. 4:**
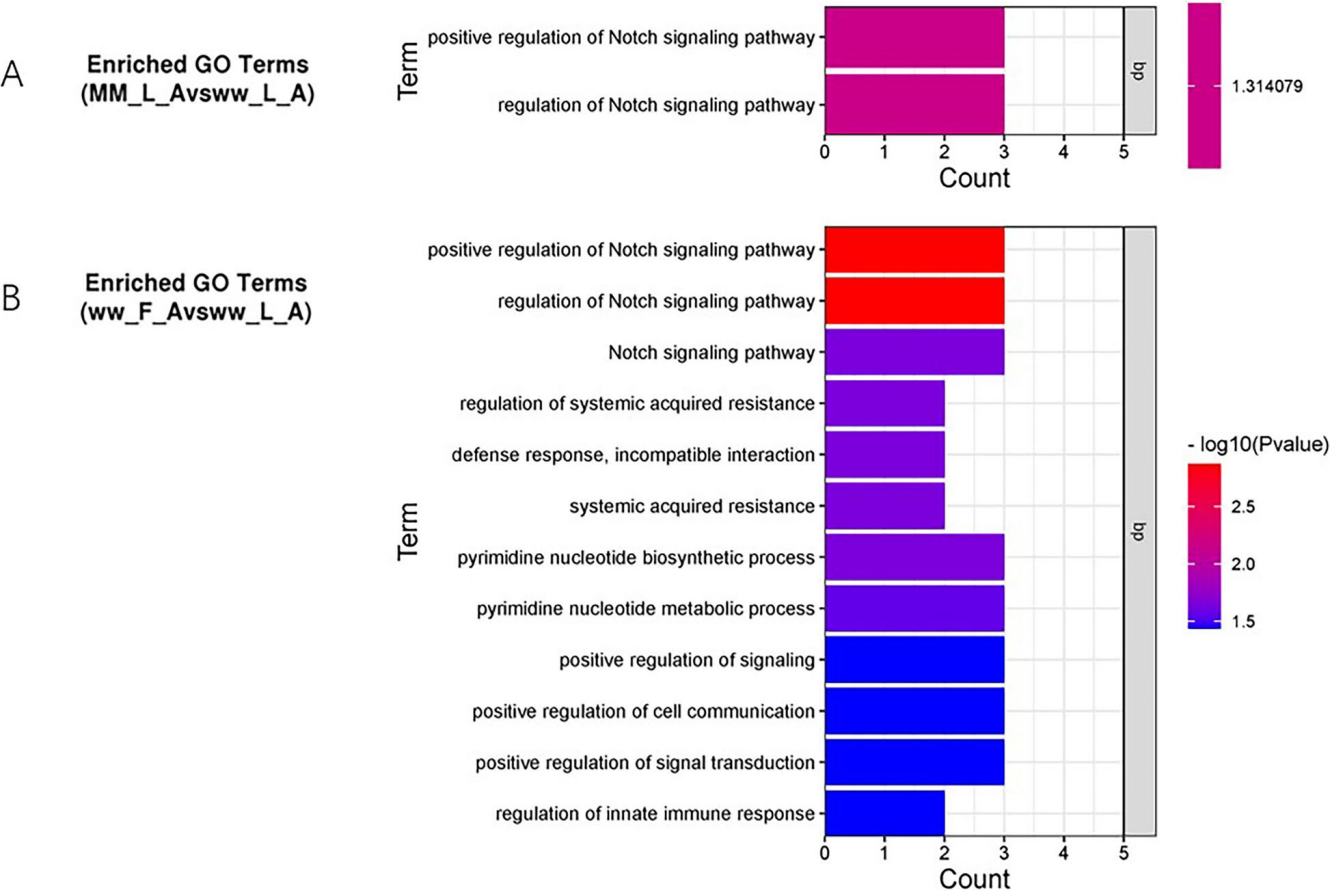
Histogram of GO enrichment of candidate target genes. Comparison of different biological processes associated to the DEMs in the MM_L_A vs. ww_L_A and ww_F_A vs. ww_L_A combinations. **A**: the DEMs according to GO enrichment analysis in MM_L_A vs. ww_L_A group. **B**: the DEMs according to GO enrichment analysis in ww_F_A vs. ww_L_A group. The x-axis indicates the number of predicted target genes enriched in the corresponding GO terms, and the color gradient indicates the *P*-value. The number of background genes was 20,364

To study the function of DEMs in the four comparisons, 4 893 and 9 318 target genes of known and novel miRNAs were predicted, respectively. Subsequently, GO term enrichment of the predicted target genes was analyzed. In the biological process category, 485, 1057 and 522 enriched GO terms were obtained from the MM_F_A vs. MM_L_A, MM_L_A vs. ww_L_A and ww_F_A vs. ww_L_A comparisons, respectively. As shown in Fig. 4, There were no enriched GO terms found in MM_F_A vs. ww_F_A. The significantly enriched GO term in the MM_L_A vs. ww_L_A comparison was the Notch signaling pathway (GO:0,008,593, P value: 0.04852), and the most significantly enriched GO terms in the ww_F_A vs. ww_L_A comparison were regulation of Notch signal transduction (GO:0,008,593, P value: 0.00135), cell communication (GO:0,010,647, P value: 0.03676), innate immune response (GO:0,045,088, P value: 0.03676), biosynthetic process and metabolic process of pyrimidine nucleotide (GO:0,006,220, P value: 0.02749; GO:0,006,221, P value: 0.02252), while there were no significantly enriched GO terms found in the MM_F_A vs. MM_L_A comparison. As the results of GO analysis showed, these DEMs have similar basic biological processes in sheep adrenal glands at different phases of estrus.

**Fig. 5:**
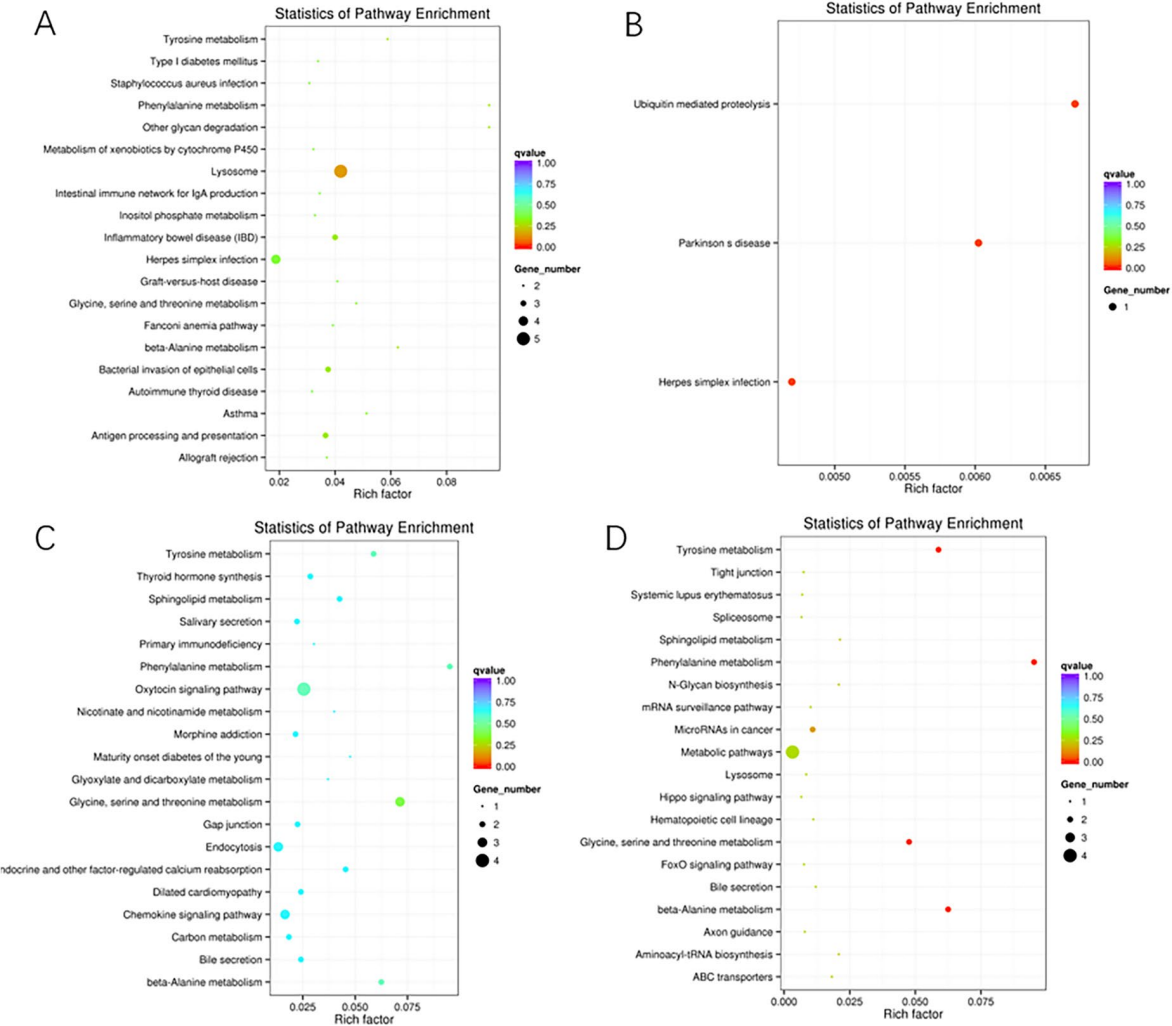
KEGG pathway enrichment analysis of DEMs. **A**: KEGG enrichment pathways for DEMs are presented for the the MM_F_A vs. MM_L_A group. **B**: KEGG enrichment pathways for DEMs are presented for the MM_F_A vs. ww_F_A group. **C**: KEGG enrichment pathways for DEMs are presented for MM_L_A vs. ww_L_A group. **D**: KEGG enrichment pathways for DEMs are presented for the ww_F_A vs. ww_L_A group. Suffixes -F and -L refer to the follicular phase and luteal phase, respectively

A total of 99, 3, 118 and 27 enriched KEGG pathways were obtained from MM_F_A vs. MM_L_A, MM_F_A vs. ww_F_A, MM_L_A vs. ww_L_A and ww_F_A vs. ww_L_A comparisons, respectively (shown in Fig. 5). The KEGG analysis of the target genes revealed several significantly enriched pathways including ubiquitin-mediated proteolysis (oas04120), Parkinson's disease (oas:101,117,250) and Herpes simplex infection (oas:101,103,036) in MM_F_A vs. ww_F_A comparison, phenylalanine metabolism (oas:101,112,335), beta-alanine metabolism (oas:101,112,335), tyrosine metabolism (oas:101,112,335), glycine, serine and threonine metabolism(101,112,335) in ww_F_A vs. ww_L_A comparison, while there were no significantly enriched pathways found in the MM_F_A vs. MM_L_A and MM_F_A vs. ww_L_A comparisons. All the results indicated that the metabolic pathway plays a pivotal role in the development and progression of the interaction between HPA and HPG.

### miRNA and mRNA coexpression network analysis

**Fig. 6:**
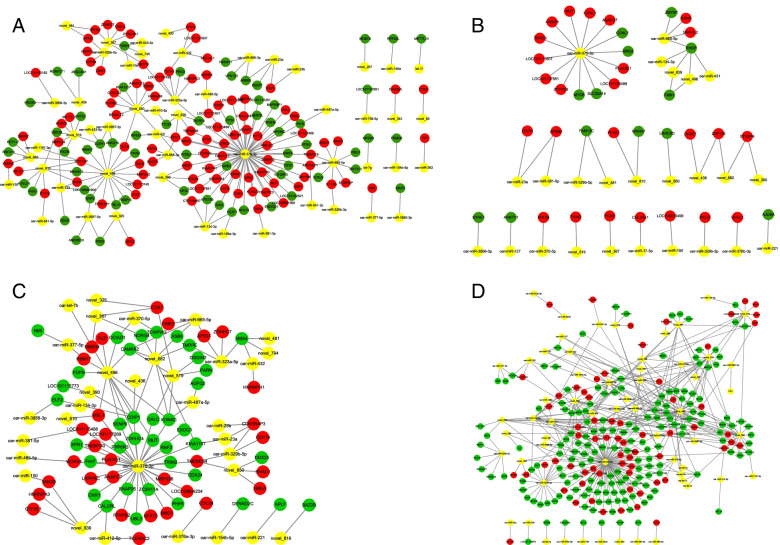
miRNA-mRNA interaction networks. The yellow node indicates miRNAs. Red indicates up-regulated genes, green indicates down-regulated genes. **A**: Network between miRNAs and it’s targets genes in the adrenal glands of MM_F_A vs. MM_L_A group. **B**: Network between miRNAs and it’s targets genes in the adrenal glands of ww_F_A vs. ww_L_A group. **C**: Network between miRNAs and it’s targets genes in the adrenal glands of MM_F_A vs. ww_F_A group. **D**: Network between miRNAs and it’s targets genes in the adrenal glands of MM_L_A vs. ww_L_A group

Combined with our previous study, the miRNA-mRNA interaction networks of the four comparison groups were constructed (shown in Fig. 6) [24]. The 38 miRNAs and 151 differentially expressed target genes were used to construct a network of MM_F_A vs. MM_L_A comparison groups. Fourteen miRNAs and 32 differentially expressed target genes were used to construct a network of ww_F_A vs. ww_L_A comparison groups. Twenty-one miRNAs and 65 differentially expressed target genes were used to construct a network of MM_F_A vs. ww_F_A comparison groups. Forty miRNAs and 233 differentially expressed target genes were used to construct a network of MM_L_A vs. ww_L_A comparison groups. Interestingly, there were 17 miRNAs in the network of both the MM_F_A vs. MM_L_A and ww_F_A vs. ww_L_A comparison groups, indicating that these miRNAs may play a crucial role in transference during the follicle phase to the luteal phase and may regulate the development and reproduction of sheep in adrenal glands.

### Small RNA-Seq data validation by quantitative polymerase chain reaction (qPCR)

**Fig. 7:**
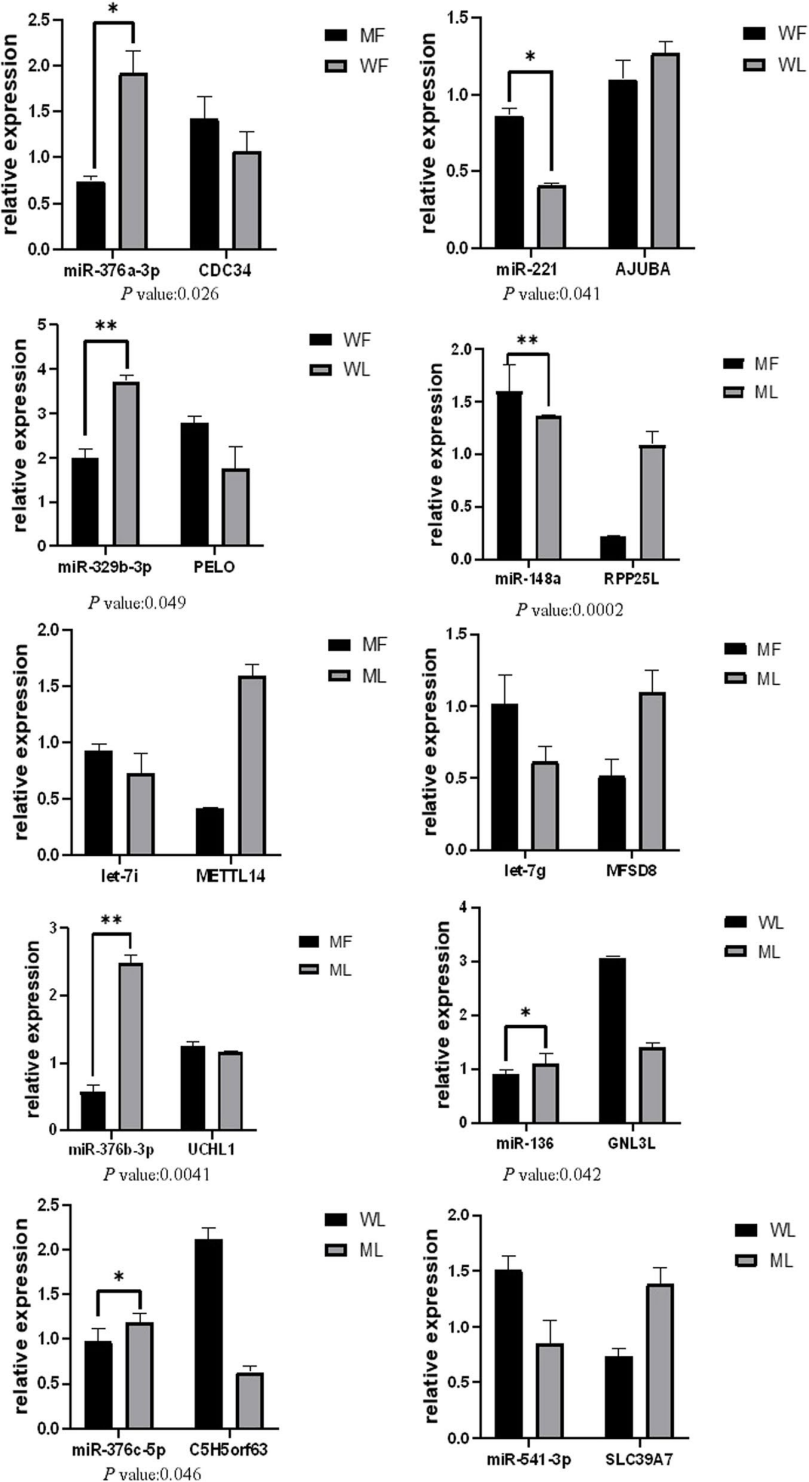
RT-qPCR verification of differentially expressed miRNA and target genes. ww_F_A and MM_F_A refer to the follicle phase of FecB +  + genotype sheep and FecB BB genotype sheep, respectively. ww_L_A and MM_L_A refer to the luteal phase of FecB +  + genotype sheep and FecB BB genotype sheep, respectively. Note: *(*P* < 0.05); **(*P* < 0.01)

To further verify the accuracy of the sequencing data, reverse-transcription qPCR (RT-qPCR) was conducted to detect the expression levels of 10 miRNA-mRNA pairs selected randomly(Table with Fold change and p-value for differentially expressed miRNAs were provided in the supplementary materials). As shown in Fig. 7, oar-miR-148a, oar-let-7i and oar-let-7 g were upregulated in the adrenal glands of MM sheep, and oar-miR-376b-3p was downregulated in MM sheep. Oar-miR-221 was upregulated and oar-miR-329b-3p was downregulated in ww sheep. The corresponding target genes (CDC34, AJUBA, PELO, RPP25L, METTL14, MFSD8, UCHL1, GNL3L, C5H5orf63, SLC39A7) of these miRNAs showed the opposite trend. These results were consistent with the transcriptome data.

## Discussion

MiRNAs, as inhibitors, play pivotal roles in the regulation of life at the gene expression and posttranslational levels [25]. In many tissues of different animals, miRNAs have an impact on the regulation of the reproductive process. In studies of donkeys, roosters, and Chinese concave-eared torrent frogs, miRNAs were found to regulate germ cells at the posttranscriptional level [26–28]. Mammal reproduction is regulated by complex hormonal and molecular networks, among which the HPA axis plays a critical role in regulating reproductive function [29]. In the brain, the structures of the HPA and HPG axes (which is the most fundamental regulatory mechanism in animal fertility) responsible for initiation of the cascade were the same, indicating that they may have the similar structures and the possibility of their interaction [20, 30]. The feedback interactions among organs of the HPA axis control physiological and behavioral responses to stress, such as glucose storage and release, immune function, digestion and reproduction [31]. Corticotrophin releasing factor (CRF), which is secreted by the paraventricular nucleus of the hypothalamus, acts on the anterior pituitary to stimulate adrenocorticotrophic hormone (ACTH) synthesis and secretion, which can ultimately stimulate the secretion of glucocorticoids from adrenal cortex cells in the adrenal glands. And the interactions between HPA axis and HPG axis have been widely reported, for example, study has shown that adrenal steroid disorders of the HPA axis could lead to the dysfunction of HPG axis, for example, deficient testosterone secretion [32], and glucocorticoids secreted by adrenal glands in HPA axis could impair the growth of ovary in HPG axis by triggering apoptosis of ovarian cells [22]. As reported, HPA axis shows the antagonism effect with the HPG axis, the overreaction of HPA axis such as stress reaction could impair the function of HPG axis, therefore results in some disease. So the maintenance of the balance between sheep reproduction and survival is necessary; therefore, as a key tissue in the HPA axis, understanding the molecular mechanisms of adrenal glands can provide insight into biomedical research and reproductive regulation in sheep. However, research on miRNAs and their targets remains to be further investigated in sheep adrenal glands.

In this study, the results of family analysis showed 10 miRNA families with more than two members, and the expression levels of these members were abundant in small-tailed Han sheep adrenal gland tissue. The most abundant miRNA was the let-7 family, seven members of which were detected in small-tailed Han sheep adrenal glands. In particular, six members, including let-7 g, let-7f, let-7i, let-7a, let-7b, and let-7c, were expressed in high abundance in different phases of different genotypes in small-tailed Han sheep, which was consistent with the characteristic of the highly conserved seed sequence among multiple let-7 isoforms [33]. Among these highly abundant miRNAs, oar-miR-148a was associated with biological processes, including cellular differentiation and development [34]. Interestingly, novel-320 was upregulated in both the MM_F_A vs. ww_F_A and MM_F_A vs. MM_L_A comparisons, indicating that novel-302 regulates its targets to influence high fertility and transform the follicle phase to the luteal phase in the adrenal glands of sheep. Researchers have found that miR-329b-3p can inhibit the proliferation and growth of tumor cells [35–39]. miR-329b-3p was downregulated in both the ww_F_A vs. ww_L_A and MM_L_A vs. ww_L_A comparisons, which illustrated that negative regulation was possible in the adrenal glands at the follicle phase and further regulated the high fertility of the FecB ++ sheep. In addition, members of the miR-376 family, including miR-376a-3p, miR-376b-3p, miR-376c-5p and miR-376d, reported to participate in the progress of growth, proliferation and metastasis of cells [40–47], were highly abundant, which showed that they may play a key role in the regulation of estrus and reproduction in sheep through adrenal glands.

In the miRNA-mRNA interaction networks of MM_F_A vs. MM_L_A comparison and ww_F_A vs. ww_L_A comparison, miR-370-3p targeted 58 mRNAs in the MM MM_F_A vs. MM_L_A group and 14 mRNAs in the ww_F_A vs. ww_L_A group, which showed the importance of miR-370-3p in the process from follicular phase transformation to luteal phase. Interestingly, there were 8 common targets of miR-370-3p between the MM_F_A vs. MM_L_A group and ww_F_A vs. ww_L_A group, including TDRD3, ANAPC7, CCNL2, LOC101107851, BRD2, LOC101111607, LOC101120489 and MUT. A previous study indicated that in stress granules, TDRD3 is located in the cytoplasm, and its Tudor domain which could recognize methylated motifs can promote survival upon treatment with chemotherapeutic drugs in cancer cells [48]. A study showed that TDRD3 was associated with the number of ovarian follicles punctured and/or oocytes retrieved. TDRD3 interacts with the FMR1 protein, resulting in insufficiency of the primary ovary [49]. A study found ANAPC7 to be a significant gene regulated by ER in ER-positive breast cancer [50]. ANAPC7 has crucial functions in cell cycle progression and eukaryotic cell mitosis [51], indicating the possibility of the effect of ANAPC7 on the reproduction of sheep. Overexpression of CCNL2 has been reported to inhibit the proliferation and differentiation of mouse embryonic carcinoma P19 cells and induce them to undergo apoptosis [52]. A study showed that BRD2 expression appears to correlate with stages of oocyte maturation [53], which confirmed its influence on sheep reproduction. A study showed that MUT deficiency induces metabolic and mitochondrial alterations, leading to cell damage [54]. In summary, we speculated that the upregulation of TDRD3, ANAPC7, LOC101107851, LOC101111607, LOC101120489 and MUT in the follicular phase, as well as the downregulation of CCNL2 and BRD2 in the follicular phase, may play roles in affecting the reproduction of sheep.

In the miRNA-mRNA interaction networks of MM_F_A vs. ww_F_A and MM_L_A vs. ww_L_A groups, miR-370-3p was also the key miRNA, which targets 34 mRNAs in MM_F_A vs. ww_F_A group and 101 mRNAs in MM_L_A vs. ww_L_A group. There were 4 identical targets of miR-370-3p: PLAC8L1, NFAT5, DDX24 and MBD1. DDX24 genes affect the development of ovaries and follicles in sheep [55]. A study showed that aberrant expression of the methylation CpG binding protein MBD1 was detected in embryonic and placental development, which reflected abnormal transcription regulation and DNA methylation involved in MBD1 [56].

The most enriched GO term was positive regulation of the Notch signaling pathway in both the MM_L_A vs. ww_L_A and ww_F_A vs. ww_L_A comparisons. As one of the conservative pathways, the effect of the Notch signaling pathway is involved mainly in cell proliferation, differentiation, apoptosis, and adhesion, especially in germ cell differentiation, to become involved in the process of growth, development, and decay in living organisms [57, 58]. PTGR2 (prostaglandin reductase 2) and LOC101103082 (enhancer of the rudimentary homolog pseudogene) were enriched in the pathways above. PTGR2 belongs to the medium-chain dehydrogenase/reductase superfamily, which can catalyze the metabolism of prostaglandin E2 [59]. A previous study showed that PTGR2 is significant in mitigating inflammatory responses [60]. As ovulation causes a local inflammatory response [11, 61], we speculated that the involvement of reproductive regulation of PTGR2 could be actualized by responding to prostaglandin, accelerating the transfer process between the follicle phase and luteal phase.

In the KEGG enrichment analysis of MM_F_A vs. ww_F_A comparison, several key genes were found to participate in the reproductive process, including UBE2R2 (ubiquitin conjugating enzyme E2 R2), CDC34 and UCHL1. Ubiquitin-conjugating enzymes play important roles in the cell cycle [62]. Previous studies have provided preliminary evidence to support that ubiquitin-conjugating enzymes CDC34 and UBE2R play an important role in ovarian development [63]. Research also showed that UBE2R2 was one of the genes in Muscovy ducks involved in the differentiation and development of the ovaries [64]. In the functional enrichment analysis of the ww_F_A vs. ww_L_A comparison, some pathways related to amino acid metabolism were significantly enriched. AOC2 (amine oxidase copper containing 2), AOC3 (membrane primary amine oxidase) and LOC101103050 (primary amine oxidase, liver isozyme) were enriched in the pathways above. The AOC2 gene encodes retina-specific amine oxidase, which was originally identified in the ganglion cell layer of the retina and has an N-terminal transmembrane segment [65]. The AOC3 gene encodes vascular adhesion protein-1, which is expressed primarily on the endothelial cell surface but also in smooth muscle cells and adipocytes [66]. Researchers have hypothesized that AOC2 evolved evolutionarily from AOC3 to become a retina-specific gene that encodes a novel adhesion protein [67]. The level of AOC3 activity has been proven to be elevated upon inflammation due to AOC3 translocation to the endothelial cell surface [68]. We speculate that the AOC2 gene plays a role in regulating the transfer of the estrus cycle via light signals. AOC3 may participate in the regulation of reproduction through local anti-inflammatory processes after ovulation. Due to the limitation of the number of the samples, the authenticity of these results was supposed to be higher but not, which means there are still further researches to be done. Explore the molecular mechanism of mRNA-miRNA on reproduction, reveal the function of mRNA/miRAN, find out plausible genes responsible for the improvement of reproduction eventually.

## Conclusions

In conclusion, the adrenal glands play a key role in sheep female reproductive processes and affect the reproduction of sheep through the hypothalamic–pituitary–adrenal axis. The adrenal glands are regulated by a variety of factors from genes to hormones. These processes are achieved by regulating different signaling pathways and related genes. In this study, we screened the DEMs (21 DEMs with 10 upregulated and 11 downregulated) and their targets (351) and constructed networks of interactions between miRNAs and mRNAs. The miRNA-mRNA pairs associated with sheep fertility were enriched by GO and KEGG analysis. Taken together, our study affirms the significance of miRNA-mRNA pathways in sheep adrenal glands. The results of the study will help us to establish a greater degree of acknowledgment of the regulatory mechanisms of miRNA-mRNA pairs in sheep reproduction.

## Materials and methods

### Ethics statement

All animals were authorized by the Science Research Department (in charge of animal welfare issues) of the Institute of Animal Sciences, Chinese Academy of Agricultural Sciences (IAS-CAAS, Beijing, China). In addition, ethical approval of animal survival was given by the animal ethics committee of IAS-CAAS (No. IAS2019-449). The study was carried out in compliance with the ARRIVE guidelines. All methods were carried out in accordance with relevant guidelines and regulations.

### Animals and sample collection

Samples used in the study were collected from small-tailed Han sheep in the Luxi area of Shandong Province, P. R. China. All sheep were fed equally under the same conditions. Healthy nonpregnant sheep aged 2 to 4 years were chosen. A total of 12 sheep (six ww and six MM) were used for the experiments. Synchronized estrus was performed on all sheep by vaginal sponges (Inter Ag Co., Ltd., New Zealand) (progesterone 300 mg) for 12 days.

Fifty hours after removing the vaginal sponges, three ww and three MM ewes were euthanized (intravenous pentobarbital (100 mg/kg)) to obtain the adrenal glands (follicular phase, ww_F_A and MM_F_A, respectively), and the remaining six sheep (three in each group) were euthanized (intravenous pentobarbital (100 mg/kg)) on the 7th day after sponge removal (luteal phase, ww_L_A and MM_L_A, respectively) to obtain the adrenal glands. All adrenal glands were immediately put into liquid nitrogen after slaughter and stored at − 80 °C for total RNA extraction. (The same animals were also used in the lncRNA and circRNA studies).

### RNA quantification and qualification

Total RNA was extracted according to the manufacturer’s instructions using TRIzol (Thermo Fisher Scientific, Waltham, MA, USA). To detect the degradation and contamination of RNA, agarose gels with a concentration of 1% were used for electrophoresis(shown in the supplementary materials). A NanoPhotometer® spectrophotometer (IMPLEN, CA, USA) was used for RNA purity detection. Qubit® RNA Assay Kit in Qubit® 2.0 A fluorometer (Life Technologies, CA, USA) was used to determine the RNA concentration. The RNA Nano 6000 Assay Kit of the Agilent Bioanalyzer 2100 system (Agilent Technologies, CA, USA) was used for RNA integrity detection.

### Library preparation for Small RNA sequencing

An RNA library was established by the input material (total amount of 3 μg total RNA per sample). Sequencing libraries were generated using NEBNext® Multiplex Small RNA Library Prep Set for Illumina® (NEB, USA.) Under the manufacturer’s recommendations, index codes were added to attribute sequences to each sample. Briefly, the 3’ ends of miRNAs, siRNAs and piRNAs were directly and specifically ligated with the NEB 3’ SR adaptor. To prevent adaptor-dimer formation, after the 3’ ligation reaction, the SR RT Primer hybridized to the excess of 3’ SR adaptor (which remained free after the 3’ ligation reaction) and transformed the single-stranded DNA adaptor into a double-stranded DNA molecule. In addition, as dsDNAs are not substrates for ligation mediated by T4 RNA ligase 1, dsDNAs do not ligate to the 5’ SR adaptor in the subsequent ligation step. First strand cDNA was synthesized by M-MuLV Reverse Transcriptase (RNase H−). LongAmp Taq 2 x Master Mix, SR Primer for Illumina and index (X) primers were used for PCR amplification. An 8% polyacrylamide gel (100 V, 80 min) was used for PCR product purification. The DNA fragments corresponding to 140–160 bp (the length of small noncoding RNA plus the 3’ and 5’ adaptors) were recovered and subsequently dissolved in 8 μL elution buffers. Finally, DNA High Sensitivity Chips were used for library quality assessment on the Agilent Bioanalyzer 2100 system. The datasets generated during the current study are available in the SRA public database repository [https://www.ncbi.nlm.nih.gov/sra/?term=PRJNA729910].

### Known miRNA alignment and novel miRNA prediction

Known miRNAs were acquired using the mapped small RNA tags. Potential miRNAs and secondary structures were obtained by modified software mirdeep2 and sRNA-tools-cli in the case of miRBase20.0 as the reference [69]. Custom scripts were used to obtain the miRNA counts as well as base bias on the first position of identified miRNA with certain length and on each position of all identified miRNA.

Novel miRNAs were predicted by the available software miREvo and mirdeep2 according to the characteristics of the hairpin of the miRNA precursor, the Dicer cleavage site and the minimum free energy of the small RNA tags unannotated in the former steps [70, 71]. At the same time, custom scripts were used to obtain the identified miRNA counts as well as base bias on the first position with a certain length and on each position of all identified miRNAs.

### miRNA family analysis

miFam.dat (http://www.mirbase.org/ftp.shtml) was used to look for families of known miRNAs. Rfam (http://rfam.sanger.ac.uk/search/) was used to look for families of novel miRNAs.

### Differential expression of miRNA

The DESeq R package (1.8.3) was used for differential expression analysis of the two groups. The *P* values were adjusted using the Benjamini and Hochberg method. A corrected *P* value of 0.05 was set as the threshold for significantly differential expression by default. The P value was adjusted using the Q-value [72]. A Q-value < 0.01 and |log2(fold change)|> 1 were set as the thresholds for significantly differential expression by default.

### Target gene prediction

Predicting the target gene of miRNA was performed by miRanda for animals [73, 74]. The miRNA expression levels were estimated by TPM (transcript per million) through the following criteria [75]: Normalization formula: Normalized expression = mapped readcount/Total reads*1,000,000.

### GO and KEGG enrichment analysis

For the target gene candidates of differentially expressed miRNAs (“target gene candidates” in the following), Gene Ontology (GO) enrichment analysis was conducted. To adjust the gene length bias, a GOseq-based Wallenius noncentral hypergeometric distribution [76] was used for GO enrichment analysis.

KEGG is a database resource for understanding the high-level functions and utilities of biological systems, such as cells, organisms and ecosystems (http://www.genome.jp/kegg/) [77–81]. KOBAS software was used to test the statistical enrichment of the target gene candidates in KEGG pathways [65].

### Construction of miRNA/mRNA networks

To investigate the functions of DEMs and their target genes in sheep prolificacy, the miRNA-mRNA network was constructed based on the data come from miRNAs and our previous study of mRNAs [25]. The diagram of the miRNA-mRNA network was generated by the tool Cytoscape v. 3.1.1 (https://cytoscape.org).

### Reverse transcription (RT)-qPCR verification

To verify the expression levels of differentially expressed miRNAs and their targets, reverse transcription and qPCR were conducted. Three samples were used for biological duplication in each group. RNA samples were the same with the samples used for RNA-seq. RT reagents (Thermo Fisher Scientific, Waltham, USA) were used for reverse transcription. With U6 small nuclear RNA as an endogenous control to normalize target gene expression, all experiments were performed in triplicate. qPCR was performed on a LightCycler 480II (Roche, Basel, Sweden) using SYBR Premix Ex Taq II. The procedure involved 40 cycles of predenaturation at 95 °C for 10 min and denaturation at 95 °C for 2 s. After the reaction was completed, melting curve analysis was performed. The relative expression levels were determined using the 2−△△Ct method [82]. The P value calculation was performed by the t-test, and *P* < 0.05 was considered to be a significant difference. The miRNA primers were designed by RiboBio Company (Guangzhou, China), and the target gene primers are shown in Table 3. PCR products on polyacrylamide gel was shown in supplementary materials.

**Table 3:** Primers of the mRNAs used in the data validation

Name	Primer sequence	Length (bp)
*CDC34*	F:5’- AAGGCACGCCTCAAGTTCC -3’ R: 5’- GGTCCTGACATTCTGCGTGG -3’	165
*AJUBA*	F:5’- CAGAGAGGACTACTTTGGCACG -3’ R:5’- GCCTCCTGAAACCCTGAAAAC -3’	201
*PELO*	F:5’- GGTACATGCCTCCTCTGGACAC -3’ R:5’- TTTCACGCTGTCCACCAGC -3’	286
*RPP25*	F:5’- AGCTGGCAGCAAGATCCG—3’ R:5’- TGGTACCCGCCGTTTGAC—3’	145
*METTL14*	F:5’- TGCAGGGCTTCCTATGATACC—3’ R:5’- CGGCCAAACCTACATCCCT—3’	271
*MFSD8*	F:5’- GAGAACATCGTGTGGATGACACA -3’ R:5’- GGTCCAGGCATACATATCCATTG -3’	206
*UCHL1*	F:5’-GCTGGGGATCTAACAGTGAAC-3’ R: 5’-AGGGTAAGGTGTCCGTTGG-3’	172
*GNL3L*	F: 5’- ACAAGGAGTTCCATAAGGTGGTG -3’ F: 5’- TGCAGCGATTCAGGTTTTTG -3’	267
*C5H5orf63*	F: 5’- ACAAAGGATCCATGTCCCCTT -3’ R:5’- TTAGCCTCCTGCACCTTGCT—3’	240
*SLC39R7*	F:5’- ACTGTCACTCTCTGGGCCTATG -3’ R: 5’- TGGCCACTGTGGGAATGTC -3’	248
*RPL19*	F: 5’-ATCGCCAATGCCAACTC-3’R:5’- CCTTTCGCTTACCTATACC—3’	154

## Supplementary Information

**Additional file 1: Supplementary Fig. 1. **Purity detection of the 12 extracted RNA samples

**Additional file 2: Supplementary Fig. 2. **PCR products of the ten mRNAs used in the data validation

**Additional file 3: Supplementary Table 1. **Readcount and TPM of each miRNA detected in the 12 samples 

**Additional file 4: Supplementary Table 2. **RQ data of miR-376c-5p

**Additional file 5: Supplementary Table 3. **Fold change and p-value for differentially expressed miRNAs

## Data Availability

The datasets generated and analyzed during the current study are available in the SRA public database [https://www.ncbi.nlm.nih.gov/sra/?term=PRJNA729910] and PRJNA729910.

## Abbreviations

MiRNA: MicroRNA
mRNA: Message RNA
DEM: Differentially expressed microRNA
GO: Gene Ontology
KEGG: Kyoto Encyclopedia of Genes and Genomes
TDRD3: Tudor domain containing 3
ANAPC7: Anaphase promoting complex subunit 7
CCNL2: Cyclin L2
BRD2: Bromodomain containing 2
MUT: Methylmalonyl-CoA mutase
PLAC8L1: PLAC8 like 1
NFAT5: Nuclear factor of activated T cells 5
DDX24: DEAD-box helicase 24
MBD1: Methyl-CpG binding domain protein 1
