# Supplementary figures and images for: miRNA-mRNA analysis of sheep adrenal glands reveals the network regulating reproduction
Source: BMC Genom Data. 2022 Jun 17;23:44. doi: 10.1186/s12863-022-01060-y (PMC9205095; doi:10.1186/s12863-022-01060-y)

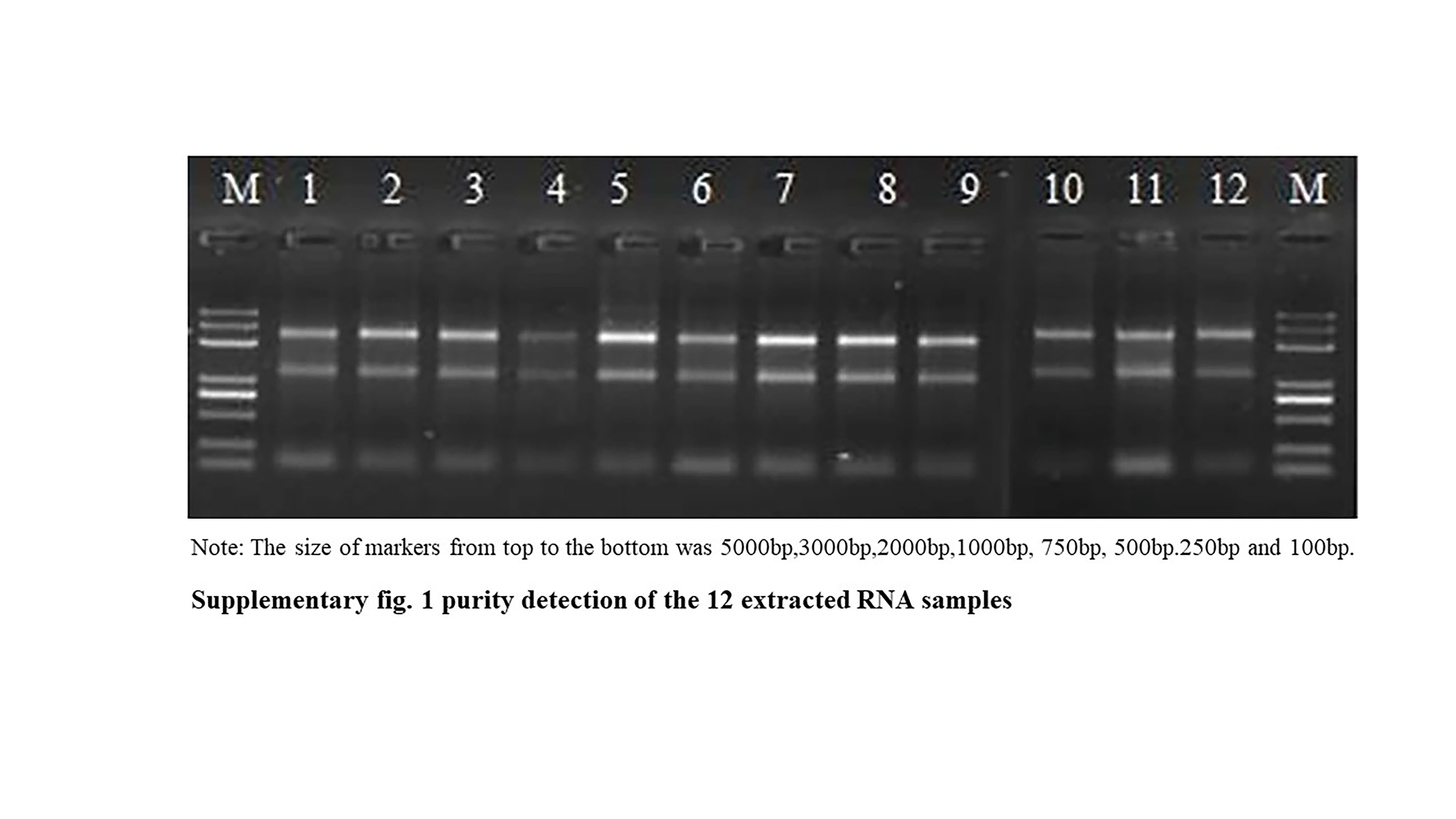

Supplement: Supplementary file 1 — Additional file 1: Supplementary Fig. 1. Purity detection of the 12 extracted RNA samples [file 12863_2022_1060_MOESM1_ESM.jpg]

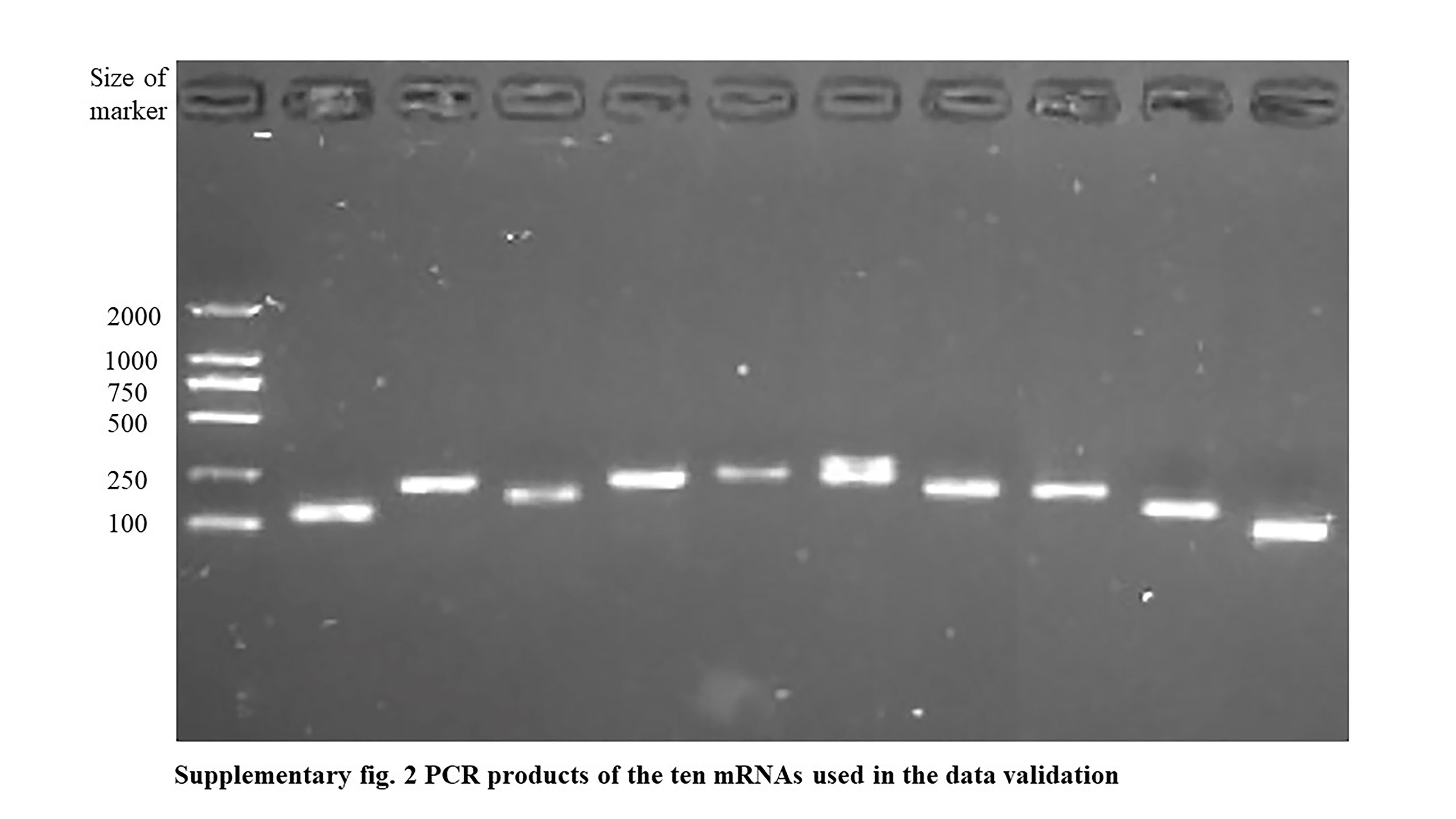

Supplement: Supplementary file 2 — Additional file 2: Supplementary Fig. 2. PCR products of the ten mRNAs used in the data validation [file 12863_2022_1060_MOESM2_ESM.jpg]
