# Supplementary material for: miRNA-mRNA analysis of sheep adrenal glands reveals the network regulating reproduction
Source: BMC Genom Data. 2022 Jun 17;23:44. doi: 10.1186/s12863-022-01060-y (PMC9205095; doi:10.1186/s12863-022-01060-y)
Supplement: Supplementary file 5 — Additional file 5: Supplementary Table 3. Fold change and p-value for differentially expressed miRNAs [file 12863_2022_1060_MOESM5_ESM.docx]

**Supplementary table 3 Fold change and p-value for differentially expressed miRNAs**

| miRNA | Fold change | P value |
| --- | --- | --- |
| miR-376a-3p | 0.39 | 0.026 |
| miR-221 | 2.10 | 0.041 |
| miR-329b-3p | 0.53 | 0.049 |
| miR-148a | 1.17 | 0.0002 |
| miR-7i | 1.27 | 0.364 |
| miR-7g | 1.645 | 0.149 |
| miR-376b-3p | 0.229 | 0.0041 |
| miR-136 | 0.824 | 0.042 |
| miR-376c-5p | 0.825 | 0.046 |
| miR-541-3p | 1.77 | 0.067 |
